# Motor imagery vividness and symptom severity in Parkinson's disease

**DOI:** 10.1111/jnp.12293

**Published:** 2022-10-13

**Authors:** Megan Rose Readman, Trevor J. Crawford, Sally A. Linkenauger, Judith Bek, Ellen Poliakoff

**Affiliations:** ^1^ Department of Psychology Lancaster University Lancaster UK; ^2^ Centre for Motor Control, Faculty of Kinesiology & Physical Education University of Toronto Toronto ON Canada; ^3^ Division of Psychology, Communication and Human Neuroscience, School of Health Sciences. University of Manchester Manchester UK

**Keywords:** bradykinesia, kinesthetic imagery, motor imagery, Parkinson's disease, visual imagery

## Abstract

Motor imagery (MI), the mental simulation of movement in the absence of overt motor output, has demonstrated potential as a technique to support rehabilitation of movement in neurological conditions such as Parkinson's disease (PD). Existing evidence suggests that MI is largely preserved in PD, but previous studies have typically examined global measures of MI and have not considered the potential impact of individual differences in symptom presentation on MI. The present study investigated the influence of severity of overall motor symptoms, bradykinesia and tremor on MI vividness scores in 44 individuals with mild to moderate idiopathic PD. Linear mixed effects modelling revealed that imagery modality and the severity of left side bradykinesia significantly influenced MI vividness ratings. Consistent with previous findings, participants rated visual motor imagery (VMI) to be more vivid than kinesthetic motor imagery (KMI). Greater severity of left side bradykinesia (but not right side bradykinesia) predicted increased vividness of KMI, while tremor severity and overall motor symptom severity did not predict vividness of MI. The specificity of the effect of bradykinesia to the left side may reflect greater premorbid vividness for the dominant (right) side or increased attention to more effortful movements on the left side of the body resulting in more vivid motor imagery.

## INTRODUCTION

Motor imagery (MI) is the mental rehearsal of an action in the absence of overt motor output (Jeannerod, 1994, 1995), which may be differentiated into visual motor imagery (VMI) and kinesthetic motor imagery (KMI) (Abbruzzese et al., 2015). VMI relates to the generation of visual representations of performing an action, while KMI relates to the sensations associated with performing an action (McAvinue & Robertson, 2008). Importantly, functional neuroimaging and lesion studies have observed that MI and motor execution activate similar cortical networks. Specifically, the primary motor cortex (Schnitzler et al., 1997; Sirigu et al., 1996) and pre‐motor areas including the supplementary motor area (SMA) (Dechent et al., 2004; Grafton et al., 1996) are activated during both overt motor output and MI (see Hardwick et al., 2018 for review). Through activating these motor areas even in the absence of overt motor output, MI can facilitate the learning of new actions (Driskell et al., 1994).

MI may also enable individuals to mentally practice actions that they are unable to perform due to physical impairments (Zimmermann‐Schlatter et al., 2008) and can facilitate safe self‐paced training in those with motor deficits (Agostini et al., 2021). Thus, MI has been identified as a potential technique for promoting the recovery of motor functioning in neurological conditions (Caligiore et al., 2017; Cuomo et al., 2022). However, MI ability may be compromised in conditions that limit movement, such as chronic pain (*e.g*. Breckenridge et al., 2019) and fibromyalgia (*e.g*. Scandola et al., 2021). In Parkinson's disease (PD), the progressive degeneration of dopaminergic nigrostriatal neurons originating in the substantia nigra pars compacta of the basal ganglia and projecting to the striatum (Agid, 1991) results in profound motor symptoms including tremor, rigidity, slowed movement execution (bradykinesia) and reduced movement amplitude (Crawford III & Zimmerman, 2011; Politis et al., 2010). Moreover, particular difficulties with voluntary, internally generated actions are observed in PD (Brown & Marsden, 1988). MI, particularly if combined with physical therapy and functional rehabilitation (Tamir et al., 2007), may be advantageous in PD neurorehabilitation by supporting the maintenance of motor capabilities (Caligiore et al., 2017), but a critical question is whether motor impairments in PD impact on MI ability (*e.g*. Poliakoff, 2013). MI has been investigated through various paradigms (McAvinue & Robertson, 2008), which can be broadly categorised as either implicit or explicit measures. Implicit MI occurs when motor representations are employed without direct instruction (Jeannerod, 1994). Hand laterality judgement tasks are widely used to assess implicit MI, whereby participants are asked to judge the laterality of images of hands presented at various angular rotations (*e.g*. Parsons, 1987a, 1987b; Ter Horst et al., 2010). The time required to make a laterality judgement in this task is proportional to the time required to physically rotate the hand into the corresponding angle (*e.g*. Parsons, 1987a). A small number of studies employing this task with people with PD have found evidence of slowing and reduced accuracy (Dominey et al., 1995; Helmich et al., 2012). However, these alterations in MI appear to parallel alterations in motor capabilities and so may be reflective of motor impairment in PD rather than an inability to perform MI (Dominey et al., 1995). Moreover, other studies have found similar performance in PD and control groups when judging hand laterality (Bek et al., 2022; Scarpina et al., 2019; van Nuenen et al., 2012).

In contrast to implicit tasks, explicit MI measures involve instructing participants to deliberately engage in MI (Jeannerod, 1994). For example, in a mental motor chronometry task, the reported time taken to imagine an action closely parallels the measured time taken to physically perform the same action (Decety et al., 1989; Milner, 1986). There is some evidence to suggest that mental chronometry may be less accurate and/or slower in individuals with PD (Scarpina et al., 2019). However, Heremans et al. (2011) found that while mental motor chronometry response times were significantly longer in individuals with PD, this slowing paralleled the slowing of their physical execution.

Self‐rating scales such as the Kinesthetic and Visual Imagery Questionnaire (KVIQ; Malouin et al., 2007) are also used as explicit measures of MI, in which participants imagine themselves performing an action and then rate the vividness of the visual image or the intensity of the kinesthetic sensations of the imagined action. Typically, healthy individuals rate VMI to be more vivid than KMI (*e.g*. Lorant & Nicolas, 2004; Malouin et al., 2007; Randhawa et al., 2010). Ratings of MI vividness in people with PD have been found to be comparable to those of healthy older adults as measured using the KVIQ (Peterson et al., 2012; Heremans et al., 2011) and the gait imagery questionnaire (Pickett et al., 2012).

Given the heterogeneity of symptom presentation and severity in PD (Lang & Lozano, 1998), it is important to consider how individual differences in symptoms may influence MI. For example, some patients present with tremor as the most dominant motor feature, whereas others never experience tremor (Greenland et al., 2019). Moreover, patients often exhibit lateralised symptom presentation (Sveinbjornsdottir, 2016). Previous studies have observed no significant effect of symptom severity, measured by overall motor scores on the Unified Parkinson's Disease Rating Scale (UPDRS; Fahn et al., 1987), on MI vividness (Heremans et al., 2011; Pickett et al., 2012), although this finding may have been affected by the exclusion of participants with severe tremor (Heremans et al., 2011). Importantly, few investigations of MI in PD have considered the influence of specific symptoms. However, one study (Helmich et al., 2012) observed that individuals with tremor made fewer errors on a hand laterality task than individuals without tremor, and this enhanced performance was coupled with increased somatosensory activation. Additionally, individuals with strongly lateralised symptoms have been found to be markedly slower in laterality judgements for images corresponding to the more affected hand (Dominey et al., 1995; Helmich et al., 2007, 2009). These findings suggest that alterations in MI may reflect alterations in motor capabilities or sensorimotor experience.

While the above findings provide important insights regarding the relative preservation of MI in PD, further investigation is needed to understand the influence of individual differences in symptom presentation and severity. For example, it is possible that particular symptoms such as tremor and bradykinesia may affect global MI measures, or that symptoms affect MI in a lateralised manner. To address this, the present study analysed the influence of overall symptom severity, tremor and bradykinesia, on MI vividness in individuals with mild to moderate PD. Moreover, potential lateralised effects of symptom severity on MI vividness were investigated by analysing the influence of side‐specific bradykinesia and tremor on side‐specific (*i.e*. left and right) VMI and KMI vividness scores.

## METHODS

### Participants

Participants were recruited through local neurology clinics and Parkinson's UK. Forty‐four participants (30 males) aged 47 to 79 years (*M* = 64.5, *SD* = 6.8) with mild to moderate idiopathic PD were included in this analysis. Based on the Edinburgh Handedness Inventory (Oldfield, 1971), 40 participants were right‐handed, 3 were left‐handed and 1 was mixed‐handed. All participants had normal or corrected‐to‐normal vision and had no history of other neurological or psychiatric conditions. Participants were screened for cognitive impairment (Addenbrookes Cognitive Examination III; Hsieh et al., 2013).

All participants except one were taking dopaminergic medication at the time of participation, including levodopa combination drugs (*e.g*. Madopar), dopamine agonists (*e.g*. Ropinirole), monoamine oxidase inhibitors (*e.g*. Rasagiline) and Catechol‐O‐Methyl Transferase (*e.g*. Entacapone).

The research was approved by a UK National Health Service (NHS) research ethics committee and participants provided written informed consent. Participants were compensated for their travel and time.

### Procedure

The data analysed here were collected as part of two previous studies (Bek et al., 2019, 2021), in which participants completed either the full (20‐item) or short (10‐item) version of the KVIQ (Malouin et al., 2007). The KVIQ has been used in several studies of MI in individuals with PD (Abbruzzese et al., 2015; Bek et al., 2019; Heremans et al., 2011; Peterson et al., 2012; Pickett et al., 2012). The KVIQ (KVIQ‐10 and KVIQ‐20; Malouin et al., 2007) has established test–retest reliability (*e.g*. Malouin et al., 2007; Randhawa et al., 2010), good concurrent validity with alternative measures of MI vividness (*e.g*. MIQ‐R; Randhawa et al., 2010) and good internal consistency (Cronbach's α KMI = .87; VMI = .89, Malouin et al., 2007).

The KVIQ requires participants to physically perform and then imagine performing, from a first‐person perspective, a series of simple actions (*e.g*. thumb‐to‐finger taps and foot tapping) involving different body parts (Malouin et al., 2007). Measures of VMI and KMI are obtained by asking participants to rate the vividness of their imagery on five‐point scales for the clarity of the visual image (VMI: 1 = no image, 2 = blurred image, 3 = moderately clear image, 4 = clear image, 5 = image as clear as seeing) and the intensity of the imagined sensations (KMI; 1 = no sensation, 2 = mildly intense, 3 = moderately intense, 4 = intense, 5 = as intense as executing the action).

The motor examination of the MDS‐UPDRS (Goetz et al., 2008) was used to assess the severity of a range of symptoms, including tremor and bradykinesia. Each item is rated on a scale of 0–4, where 0 indicates a complete absence of the symptom, and 4 indicates severe disability. Severity is assessed independently for each limb and side where applicable (*e.g*. for resting tremor).

### Data analysis

As participants had completed either the full KVIQ or the short‐form KVIQ‐10, only items from the KVIQ‐10 (Malouin et al., 2007) were included in the present analysis for all participants. The KVIQ‐10 includes several limb‐specific movements and one trunk movement. For the purpose of the present study, each of the limb‐specific actions was repeated for both sides of the body, providing a measure of VMI and KMI vividness for each body side. Internal consistency of the VMI and KMI subscales was calculated.

To analyse the influence of motor symptoms on MI at a body side‐specific level, the following KVIQ items were analysed separately for right and left limbs: forward shoulder flexion, thumb‐to‐finger tips, hip abduction and foot tapping. For the overall analysis, items from both sides, as well as forward trunk flexion were included.

From the MDS‐UPDRS (hereafter, ‘UPDRS’), overall motor scores, as well as measures of overall bradykinesia and tremor severity, and side‐specific severity of tremor and bradykinesia were calculated. For bradykinesia at a side‐specific level, the following UPDRS items were analysed separately for right and left limbs: finger tapping, hand movements, pronation‐supination of hands, toe tapping, leg agility. For the overall analysis, items from both sides were included, as well as global spontaneity of movement (bradykinesia). For side‐specific tremor, the following UPDRS items were analysed separately for right and left limbs: postural tremor of the hands, kinetic tremor of the hands, rest tremor amplitude (upper and lower limbs). For the overall analysis, items from both sides were included, as well as rest tremor amplitude for the lip/jaw and constancy of rest tremor.

Linear mixed effects modelling (LMM) was used to analyse the association of symptom severity with KVIQ‐10 scores (i) overall and (ii) at a side‐specific level. Given that healthy adults commonly rate VMI to be more vivid than KMI (*e.g*. Malouin et al., 2007; Randhawa et al., 2010), imagery modality (VMI, KMI) was also included as a predictor when analysing the effects of symptoms. LMM allows the influence of fixed effects of independent variables to be analysed, while accounting for random effects corresponding to unexplained differences such as variation between participants (Baayen et al., 2008). Models were fitted using the maximum likelihood procedure with the Satterthwaite adjustment method in the lme4 package (Bates et al., 2014) in R (R Core Team, 2021). Models were compared using likelihood ratio tests. A further analysis that included only right‐handed participants produced the same pattern of results, so all participants were included in the final analyses.

## RESULTS

### MI and motor symptoms

UPDRS motor scores and KVIQ‐10 scores are presented in Table 1. All participants had mild to moderate symptoms as indicated by the Hoehn and Yahr scale (*M* = 1.98, *SD* = 0.81), with a mean UPDRS score of 37.43 (*SD* = 9.57). Good internal consistency was found for the KVIQ subscales used within this study (KMI Cronbach's alpha = .88; VMI Cronbach's alpha = .89).

**TABLE 1:** Total and side‐specific UPDRS motor scores and total and side‐specific KVIQ‐10 scores. Minimum and maximum possible scores are provided for reference.

Measure	Possible score range	Mean score (*SD*)
Total UPDRS motor	0–132	37.43 (9.57)
Total Bradykinesia	0–48	14.80 (5.39)
Right Bradykinesia	0–20	5.55 (3.18)
Left Bradykinesia	0–20	7.89 (3.12)
Total Tremor	0–40	5.43 (4.30)
Right Tremor	0–16	1.80 (1.46)
Left Tremor	0–16	2.50 (2.28)
Total KVIQ‐10	18–90	63.52 (16.68)
Total VMI	9–45	34.18 (8.95)
Total KMI	9–45	29.34 (9.70)
Right VMI	4–20	15.34 (3.96)
Left VMI	4–20	14.84 (4.21)
Right KMI	4–20	12.84 (4.09)
Left KMI	4–20	12.39 (4.24)

#### Effects of modality and symptoms on overall MI

To examine the influence of overall symptom severity, tremor, bradykinesia and MI modality on overall MI vividness, LMM analysis was conducted with total KVIQ‐10 score (MI) as the dependent measure, modality (KMI or VMI), total UPDRS motor score, total bradykinesia and total tremor scores as fixed effects and participants as random intercepts. MI was only significantly influenced by modality, reflecting higher vividness ratings for VMI compared to KMI (*b* = 5.66, *SE* = 1.21, *t*[44] = 4.67; *p* < .001).

In a subsequent model, total tremor and bradykinesia scores were replaced with side‐specific tremor and bradykinesia scores. KVIQ scores were predicted by modality, again reflecting higher vividness ratings for VMI compared to KMI (*b* = 5.66, *SE* = 1.21, *t*[44] = 4.67; *p* < .001) and by left side bradykinesia (*b* = 1.54, *SE* = .59, *t*[44] = 2.64; *p* = .011), such that higher bradykinesia scores for the left side of the body were associated with higher MI vividness ratings.

Comparison of the two models revealed no significant difference (χ^2^[2] = 5.15; *p* = .076). Moreover, removing all non‐significant predictors from the original model did not significantly affect the fit of the model (χ^2^[3] = 4.65; *p* = .20), such that the best‐fitting model included only the random intercept for participants and the fixed effect of modality (see Table 2).

**TABLE 2:** Summary of best‐fitting linear mixed‐effect models analysing the effects of modality (visual vs. kinesthetic) and symptoms (UPDRS motor scores) on motor imagery (KVIQ‐10) scores overall and for left and right sides of the body.

Model	Predictors (*b*, *SE*, *df*, *t*, *p*)	Model *df*	BIC	AIC	LogLik	Deviance	Marginal/conditional R2
Total KVIQ‐10		84	623.7	633.6	−307.9	615.7	.09/.63
(Intercept)	25.21, 5.35, 45.14, 4.71, <.001						
Modality: Visual	5.66, 1.21, 44, 4.67, <.001						
Left side KVIQ		83	491.5	503.9	−240.8	481.5	.16/.57
(Intercept)	9.26, 1.46, 47.98, 6.34, <.001						
Modality: Visual	2.45, .61, 44, 4.03, <.001						
Bradykinesia_ Left	.40, .17, 44, 2.35, .023						
Right side KVIQ		84	479.7	489.6	−235.8	471.7	.09/.65
(Intercept)	12.84, .60, 63.70, 21.40, <.001						
Modality: Visual	2.50, .52, 44, 4.77, <.001						

#### Effects of modality and symptoms on side‐specific MI

**FIGURE 1:**
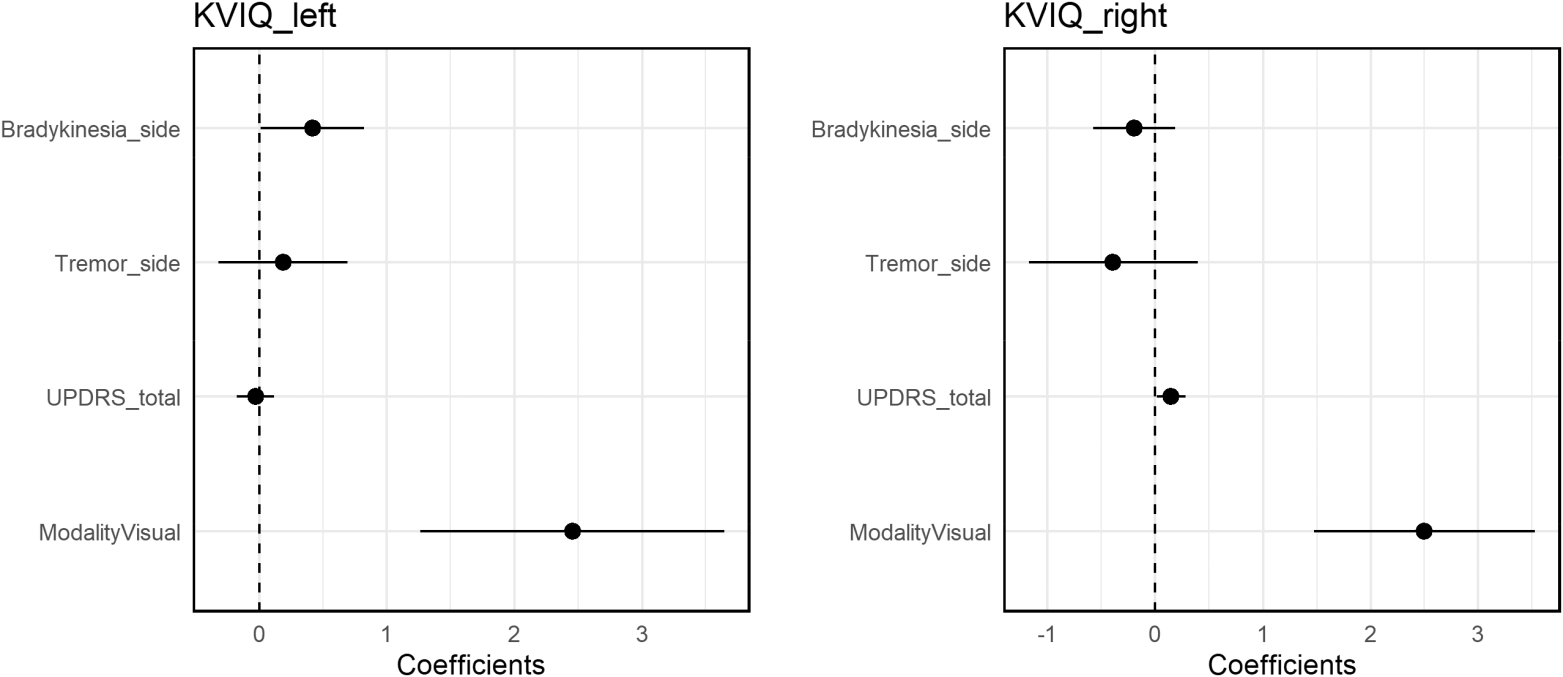
Dot‐and‐whisker plots (coefficients and 95% CIs) showing prediction of left and right side MI (KVIQ) scores by imagery modality (visual vs. kinesthetic), UPDRS total motor score and side‐specific bradykinesia and tremor. For the left side, MI score was best predicted by modality and bradykinesia, while right side MI was best predicted by modality alone.

KVIQ scores for left and right side movements were analysed in separate models, with modality (VMI or KMI), UPDRS total motor score, side‐specific bradykinesia and side‐specific tremor as fixed effects and random intercept effects of participants. For left side MI, modality (*b* = 2.45, *SE* = .61, *t*[44] = 4.03; *p* < .001) and left side bradykinesia (*b* = .42, *SE* = .21, *t*[44] = 2.00; *p* = .045) were significant. Removing all non‐significant predictors did not affect the model fit (χ^2^[2] = .53; *p* = .77), and the model including both modality and left side bradykinesia was superior to models with modality alone (χ^2^[1] = 5.21; *p* = .022) or bradykinesia alone (χ^2^[1] = 13.84; *p* < .001) (Table 2). As illustrated in Figure 1, VMI (vs. KMI) and higher left side bradykinesia scores were associated with higher vividness scores. For right side MI, modality (*b* = 2.50, *SE* = .52, *t*[44] = 4.77; *p* < .001) and UPDRS total motor score (*b* = .15, *SE* = .068, *t*[44] = 2.17; *p* = .035) were significant. Excluding all non‐significant predictors did not significantly affect the model fit (χ^2^[3] = 4.56; *p* = .21); moreover, removing UPDRS score did not significantly reduce the model fit (χ^2^[1] = 2.34; *p* = .13), indicating that the model including modality only provided the best fit (Table 2). Again, vividness scores were higher for VMI than KMI (see Figure 1).

**FIGURE 2:**
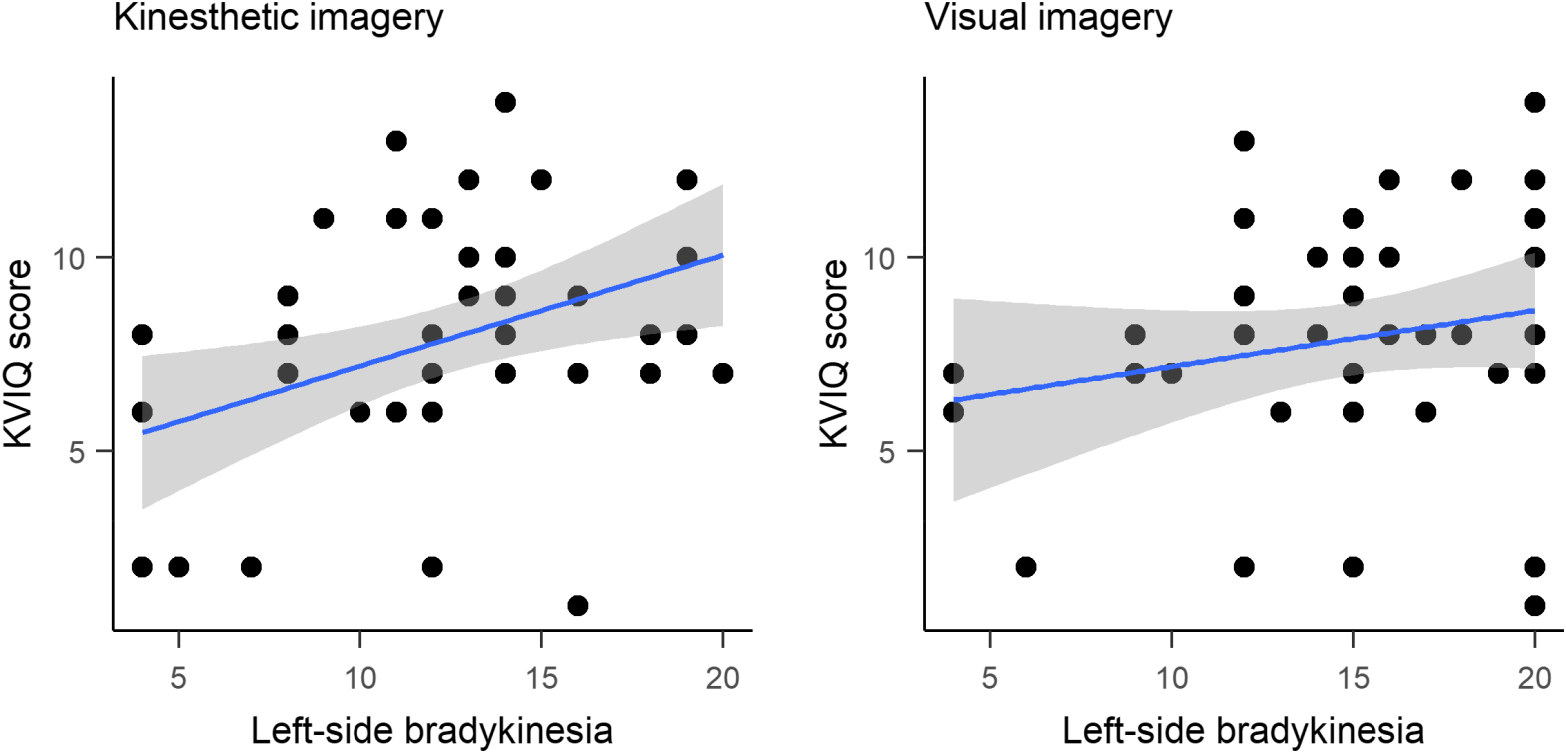
Scatterplots showing the correlation between left side bradykinesia and left side KVIQ scores, which was significant for KMI (left) but not VMI (right).

The relationships between left side bradykinesia and VMI and KMI scores for the left side were further explored using Spearman correlation coefficients (see Figure 2). There was a significant positive association between left side KMI and left side bradykinesia (*rs*[40] = .31; *p* = .042) but the association between left side VMI and left side bradykinesia was not significant (*rs*[40] = .20; *p* = .20).

## DISCUSSION

The present study examined the influence of motor symptom type and lateralisation on MI vividness in individuals with mild to moderate PD. While MI vividness was not associated with overall motor symptom severity or tremor, greater severity of left side bradykinesia was associated with increased vividness of kinesthetic MI for the left side of the body. Additionally, participants with PD reported greater vividness of VMI than KMI, consistent with previous findings from healthy participants (*e.g*. Bek et al., 2019; Lorant & Nicolas, 2004; Malouin et al., 2007; Randhawa et al., 2010).

Although tremor is a common symptom of PD, approximately 30% of individuals with PD do not experience tremor (Crawford III & Zimmerman, 2011). In comparison, almost all individuals with PD experience some degree of bradykinesia (Chaudhuri & Ondo, 2011). It has been proposed that bradykinesia occurs as a result of the failure of basal ganglia output to stimulate cortical mechanisms associated with the preparation and execution of actions (*e.g*. Berardelli et al., 2001). This is supported by electrophysiological evidence showing that the spatiotemporal pattern of movement related desynchronisation preceding voluntary movement is delayed in untreated PD patients, indicating that motor preparation is impaired (Defebvre et al., 1996).

Several studies have observed that the cortical activity of MI substantially overlaps with the cortical activity during motor planning (Jeannerod, 2001; Lotze & Halsband, 2006; Monaco et al., 2020). For example, the dorsolateral prefrontal cortex and corresponding regions of the frontal thalamus are recruited in both motor preparation and MI but not motor execution (Hardwick et al., 2018). This has subsequently led to the proposal that MI is more closely related to motor planning than to motor execution (Toovey et al., 2020; Toussaint et al., 2013).

Parkinsonian tremor is thought to arise as a consequence of aberrant neural oscillations within the cortico‐basal ganglia‐thalamic neural circuits (Singh, 2018). While some studies have observed relationships between low frequency oscillatory activity in the SMA and the onset of voluntary action in healthy individuals (Armstrong et al., 2018; Schmidt et al., 2016), these studies have not determined whether such oscillatory activities have a causal role in motor planning and initiation or are a by‐product of in motor planning and initiation (Armstrong et al., 2018). Furthermore, the relationship between the oscillatory activity associated with tremor and motor planning and initiation in PD is still largely unknown. As a result, the different neurophysiology of tremor and bradykinesia and their relationship to motor planning could potentially explain why bradykinesia and tremor may differentially influence MI.

Although the present study did not find a significant influence of tremor on vividness of MI, Helmich et al. (2012) found that increased tremor was associated with reduced error in a hand laterality task. Therefore, the influence of specific symptoms on MI may differ according to how MI is assessed. Based on principal components analysis, it has been proposed that the generation, maintenance and manipulation of MI represent distinct dimensions of MI (Kraeutner et al., 2020); in particular, the hand laterality task was suggested to involve the maintenance and manipulation of MI, whereas the KVIQ was suggested to involve generation of MI. Moreover, Saimpont et al. (2015) found that MI vividness, measured using the KVIQ‐10, did not significantly correlate with measures of MI manipulability (finger‐thumb opposition task) or motor chronometry. Further analyses directly comparing the influence of specific PD symptoms on multiple measures of MI would there be informative.

Further, the KVIQ requires participants to perform an action prior to imagining the performance of this action. Thus, it is possible that the physical performance of the action influences MI vividness. However, several studies have observed symptom/effector‐specific effects on hand laterality judgement (Dominey et al., 1995; Helmich et al., 2007), suggesting that MI can be influenced by PD symptoms even without a physical movement component to the task. Future studies could further investigate the influence of PD symptoms on MI tasks that do not involve a physical component.

Moreover, MI may be generated from either a first‐person or third‐person (*i.e*. as if looking at someone else) perspective (Isaac et al., 1986; Roberts et al., 2008). Investigations of co‐speech gesture (Humphries et al., 2016) and body representation (Conson et al., 2014) in PD suggest that people with PD may have an increased tendency to represent actions from the third‐person perspective, which may reflect a difficulty in adopting a first‐person perspective (De Bellis et al., 2017; Saxe et al., 2006). Thus, it is possible that PD symptoms influence first and third person MI differently, and this should be explored in further research.

The present study is the first to demonstrate a specific influence of left side bradykinesia on MI, but the mechanisms underlying this relationship are yet to be determined. One possible explanation for this finding focuses on the cortical lateralisation of MI. In PD, lateralised symptoms are reflective of dopaminergic degeneration and uptake in the contralateral substantia nigra and putamen (Choe et al., 1998; Lin et al., 2014; Wang et al., 2015), such that left side bradykinesia reflects disruption in the right basal ganglia.

While the lateralisation of MI is not yet fully understood, some evidence suggests that KMI may be more lateralised to the right hemisphere (Ehrlichman & Barrett, 1983). For example, Lebon et al. (2018) found that when healthy participants imagined performing a finger tapping sequence, particularly high levels of KMI were associated with strong activation of the right inferior parietal lobe. Similarly, Zabicki et al. (2019) found a significant correlation between KMI vividness and right inferior and superior parietal lobe activation. Therefore, if KMI is a right parietal function (Lebon et al., 2018; Zabicki et al., 2019), then we might anticipate that left side bradykinesia would influence MI to a greater extent than right side bradykinesia.

It additionally, previous research has indicated that while right‐lateralised symptoms are associated with language and verbal memory deficits, left‐lateralised symptoms are associated with spatial attention, visuospatial functions and mental rotation deficits (Verreyt et al., 2011). For example, visual imagery scores, assessed by the Vividness of Visual Imagery Questionnaire and Test of Visual Imagery Control, and VMI assessed through mental rotation tasks, were found to be poorer in the presence of predominantly left side lateralised symptoms in PD (Monaco et al., 2018; Verreyt et al., 2011). Conversely, KMI as measured by the Vividness of Movement Imagery Questionnaire was not found to be influenced by left side lateralised symptoms (Monaco et al., 2018), although different symptoms such as bradykinesia and tremor were not analysed separately. It should be noted, however, that our findings as well as these previous findings relate to lateralised symptoms (more prominent in one side of the body) rather than purely unilateral symptoms.

Another possibility is that the specific influence of left side bradykinesia on MI relates to hand dominance. Most of the participants in the present study (93%) were right‐hand dominant. In healthy right‐handed individuals, KMI is found to be more vivid for the dominant hand than the non‐dominant hand (Matsuo et al., 2020). The absence of an effect of right side bradykinesia in the present study may, therefore, reflect the tendency for more vivid imagery for the dominant side of the body, such that it is more resistant to symptomatic effects.

Moreover, as the physical performance of left‐sided movement is more difficult for right‐dominant individuals (Incel et al., 2002; Judge & Stirling, 2003), it may be that bradykinesia in the left side increases attention to movements on that side as they become slower and more effortful than usual. This account would be consistent with previous research that found MI to be slowed in accordance with motor execution in PD (Conson et al., 2014; Dominey et al., 1995; Heremans et al., 2011) and evidence that MI can show lateralised effects in PD (Conson et al., 2014; Dominey et al., 1995; Helmich et al., 2007).

In summary, the present study demonstrated that in people with mild to moderate PD, similar to healthy participants, vividness was greater for VMI than for KMI, and more severe left side bradykinesia was associated with more vivid KMI. The difference in  influence of bradykinesia and tremor on MI may be due to the different neurophysiology underlying these symptoms. Moreover, greater premorbid vividness of KMI for the dominant body side, and increased effort and slowing of movements in the non‐dominant side, may explain the increased vividness of KMI with increased left side bradykinesia. These findings indicate that MI may differ between body sides in accordance with differences in symptomatology. While further research is needed to replicate and extend these findings, such differences should be taken into consideration when designing MI‐based interventions for people with PD.

## AUTHOR CONTRIBUTIONS

**Megan Rose Readman:** Conceptualization; formal analysis; funding acquisition; investigation; methodology; project administration; resources; visualization; writing – original draft; writing – review and editing. **Trevor J. Crawford:** Conceptualization; supervision; writing – review and editing. **Sally A. Linkenauger:** Conceptualization; supervision; writing – review and editing. **Judith Bek:** Conceptualization; data curation; formal analysis; funding acquisition; methodology; resources; software; supervision; visualization; writing – review and editing. **Ellen Poliakoff:** Conceptualization; data curation; formal analysis; funding acquisition; supervision; visualization; writing – review and editing.

## CONFLICT OF INTEREST

All authors declare no conflict of interest.

## Data Availability

The data that support the findings of this study are openly available in figshare at https://doi.org/10.6084/m9.figshare.19182020.v1.
